# Mapping the Nitric Oxide Axis in IVF: Genotype Associations in Antagonist Cycles

**DOI:** 10.3390/ijms262211187

**Published:** 2025-11-19

**Authors:** Charalampos Voros, Fotios Chatzinikolaou, Georgios Papadimas, Spyridon Polykalas, Despoina Mavrogianni, Aristotelis-Marios Koulakmanidis, Diamantis Athanasiou, Vasiliki Kanaka, Maria Kanaka, Kyriakos Bananis, Antonia Athanasiou, Aikaterini Athanasiou, Ioannis K. Papapanagiotou, Charalampos Tsimpoukelis, Maria Anastasia Daskalaki, Marianna Theodora, Nikolaos Thomakos, Panagiotis Antsaklis, Georgios Daskalakis, Dimitrios Loutradis

**Affiliations:** 1 1st Department of Obstetrics and Gynecology, ‘Alexandra’ General Hospital, National and Kapodistrian University of Athens, 80 Vasilissis Sofias Avenue, 11528 Athens, Greece; depy.mavrogianni@yahoo.com (D.M.); aristoteliskoulak@gmail.com (A.-M.K.); vasilikikanaka@outlook.com (V.K.); maira.kanaka24@gmail.com (M.K.); tsimpoukelischa@gmail.com (C.T.); md181341@students.euc.ac.cy (M.A.D.); martheodr@gmail.com (M.T.); thomakir@hotmail.com (N.T.); panosant@gmail.com (P.A.); 2 Laboratory of Forensic Medicine and Toxicology, School of Medicine, Aristotle University of Thessaloniki, 54124 Athens, Greece; fotischatzin@auth.gr (F.C.); loutradi@otenet.gr (D.L.); 3 Athens Medical School, National and Kapodistrian University of Athens, 15772 Athens, Greece; dr.georgepapadimas@gmail.com (G.P.); spyridonpolykalas@hotmail.com (S.P.); gpapamd@hotmail.com (I.K.P.); 4 IVF Athens Reproduction Center, 15123 Maroussi, Greece; diamathan16@gmail.com (D.A.); antoathan16@gmail.com (A.A.); diamathan17@gmail.com (A.A.); 5 King’s College Hospitals NHS Foundation Trust, London SE5 9RS, UK; kyriakos.bananis@nhs.net; 6 Fertility Institute-Assisted Reproduction Unit, Paster 15, 11528 Athens, Greece

**Keywords:** endothelial nitric oxide synthase, NOS3 polymorphism, rs1799983 (Glu298Asp), nitric oxide, follicular fluid, β-hCG, ovarian stimulation, IVF, oocyte competence, gonadotropin response

## Abstract

Endothelial nitric oxide synthase (eNOS, NOS3) regulates steroidogenesis, redox signalling, and the vascular tone of the ovaries. Despite varying outcomes in previous studies, the prevalent NOS3 rs1799983 (Glu298Asp) polymorphism may influence endocrine function during controlled ovarian stimulation (COS). On the retrieval day, we assessed follicular-fluid hormones, day-3 hormones, and controlled ovarian stimulation (COS) outcomes (follicles, oocytes, MII oocytes, embryos) in 62 antagonist IVF/ICSI cycles classified by NOS3 genotype (GG/GT/TT). The outcomes for COS and early-cycle hormones were mostly consistent across all genotypes. A similar allele-dose pattern was seen for baseline oestradiol (GG < GT < TT), with heterozygous carriers displaying higher levels of follicular-fluid β-hCG relative to GG individuals. No changes were seen in follicle count, oocyte production, nuclear maturation, or embryo development. Baseline oestradiol and follicular β-hCG serve as the principal indications of the modest, context-dependent endocrine effects of the NOS3 rs1799983 polymorphism in antagonist cycles. To clarify the clinical significance of these intricate genotype-associated patterns, additional comprehensive, genotype-balanced investigations that include direct NO-pathway phenotyping are essential.

## 1. Introduction

eNOS is a crucial enzyme responsible for the production of NO, a gas that regulates leukocyte-endothelial interactions, tissue perfusion, platelet inactivity, vasodilation, and redox-sensitive signalling across organ systems [1]. Nitric oxide is not only a passive element in the control of vascular tone inside the ovary; it infiltrates the follicular milieu, affecting luteal vascularization, cumulus-oocyte complex development, granulosa and theca cell functioning, and peri-ovulatory remodelling [2]. When NO is dysfunctional, it reacts with superoxide to form peroxynitrite, resulting in nitrosative stress, protein tyrosine nitration, DNA damage, and mitochondrial dysfunction. At healthy concentrations, nitric oxide activates soluble guanylyl cyclase, increases cGMP levels, and modifies Ca2+ fluxes and kinase cascades (e.g., PI3K/Akt, PKG) that govern steroidogenesis and follicular growth [3].

From a molecular pathology standpoint, common variations in NOS3, including coding substitutions (e.g., Glu298Asp/rs1799983), promoter modifications (e.g., T−786C), and intron-4 VNTRs (4a/4b), have been associated with altered transcriptional activity, variations in proteolytic vulnerability, and an increased likelihood of eNOS uncoupling under oxidative stress in diverse tissues [4]. Uncoupled eNOS generates superoxide instead of nitric oxide by altering the electron flow to decrease O_2_ rather than L-arginine. This diminishes the quantity of accessible nitric oxide and elevates oxidative stress [5]. Upstream modulators, such as endothelial arginase (which competes for substrates), the endogenous NOS inhibitor ADMA (regulated by DDAH enzymes), and the availability of BH4 (regulated by GTP cyclohydrolase and salvage pathways), exacerbate the imbalance between coupled and uncoupled states. In the follicle, nitric oxide-sensitive nodes in granulosa and cumulus cells (e.g., ERK1/2, cAMP/PKA cross-talk) may alter steroidogenesis, cumulus cell proliferation, and the resumption of meiosis [6]. The ovarian vasculature’s shift towards uncoupling may diminish microvascular tone, impede angiogenesis, and weaken endothelial barrier function. These mechanisms promote a study design that accounts for redox context and subtle genotype effects while reducing dependence on parametric assumptions, and they offer a cohesive framework for understanding how NOS3 variation may affect both COS performance and endocrine measurements on cycle day 3 [7].

The clinical evidence linking NOS3 polymorphisms to reproductive endocrinology and IVF outcomes has been persuasive but inconsistent, attributable to discrepancies in sample size, ancestral background, stimulation protocols (agonist vs. antagonist; recombinant vs. urinary gonadotropins; source of LH bioactivity), and definitions of endpoints (oocytes retrieved vs. MII oocytes vs. viable embryos or rates) [8].

Day 3 (early follicular) hormones provide mechanistic insights into upstream endothelial and redox biology, while also being crucial for assessing ovarian reserve and predicting stimulation response [9]. The present research investigates the relationship between NOS3 genetic variation and hormonal profiles in endocrine and follicular fluid that may indirectly reflect NO-axis regulation during controlled ovarian stimulation, instead of directly quantifying nitric oxide metabolites. E2 measures basal aromatase activity and granulosa function; PRL affects GnRH pulsatility and gonadotropin efficacy; FSH and LH reflect hypothalamic-pituitary set points and ovarian feedback mechanisms; and AMH examines the small antral follicle reservoir and corresponds with recruitable follicles [10]. signifies an inflammatory milieu that might exacerbate endothelial dysfunction via cytokines and reactive oxygen species (ROS), factors that Thyroid autoimmunity (anti-TPO, anti-TG)facilitate eNOS uncoupling [11].

Clinically relevant goals that cover endocrine sufficiency, vascular support, and cellular fitness throughout the stimulation cycle include follicle count, retrieved oocytes, mature MII oocytes, embryos, and their corresponding rates (MII_rate, Embryo_rate) [12]. A minor deficiency of nitric oxide might result in reduced estradiol levels per follicle (less efficient activation of granulosa aromatase), diminished follicular recruitment (due to compromised stromal microcirculation), or inadequate cytoplasmic maturation (mitochondrial and spindle stress). All of these factors may diminish MII yield or embryonic development [2,13]. Conversely, excessive or misaligned NO signals may impair checkpoint regulation and spindle formation, thereby impacting euploidy potential. Thus, these findings act as informative indicators of genotype-related mechanisms: if NOS3 variation affects NO bioavailability or eNOS coupling status, the signal should manifest as small-to-moderate, consistent changes across these metrics, even when individual *p*-values near conventional thresholds due to low TT counts [14,15].

To examine these relationships in a practical setting, we analysed a single-centre cohort of 62 women undergoing IVF/ICSI, treated with one of three standard stimulation protocols: human menopausal gonadotropin (hMG; *n* = 16), recombinant FSH (rFSH; *n* = 25), or rFSH in conjunction with hCG to stimulate LH activity (*n* = 21). The pharmacodynamics and source of LH bioactivity vary across various regimens; exogenous hCG interacts with the LH receptor, displaying a unique kinetic profile in contrast to LH in hMG. These disparities may affect endothelial eNOS activation, corpus luteum neovascularization, and angiogenic signalling pathways, including VEGF/angiopoietin pathways [16]. The strategy reduces allocation bias while reflecting typical decision-making processes, thereby enhancing external validity, since protocol allocation was based on clinical factors (age, AMH/FSH levels, past response) instead of genotype [17]. LH influences VEGF expression and endothelial interactions, so enabling the investigation of whether genotype effects are more pronounced in certain LH-activity situations (e.g., hCG-rich vs. LH-derived), which serves as a physiologically realistic modifier [18]. Thus, NOS3 polymorphisms may influence nitric oxide bioavailability and microvascular function in subtle, context-dependent ways, thus impacting early-cycle endocrine control. Thus, the current investigation investigates whether these genotype-specific variances result in observable differences in the efficacy of controlled ovarian stimulation and day-3 hormone levels.

Our main aim is to determine the correlation between the eNOS polymorphism and (a) the day-3 hormonal profile and (b) COS outcomes across GG/GT/TT genotypes. We are specifically concentrating on an additive dosage trend from GG to GT to TT that illustrates allele-dose biology. This multi-axis overview of endocrine status may identify influences from upstream endothelial and redox sources. The hormonal panel includes FSH, LH, PRL, AMH, TSH, T3, T4, FT3, FT4, anti-TPO, anti-TG, E2, testosterone, and Δ4-androstenedione. The clinical outcomes include follicles, eggs, mature MII, embryos, and the resultant MII_rate and Embryo_rate, which encapsulate recruitment, retrieval, nuclear/cytoplasmic maturation, and early developmental competence. We investigate whether genotype-associated patterns related to eNOS coupling and uncoupling biology result in consistent endocrine changes and performance fluctuations throughout COS, despite the anticipation of minor impacts presumably affected by the surrounding hormonal and immunological milieu.

## 2. Results

We start by outlining the group of people who are starting COS. Comprehensive descriptive statistics for demographics and cycle-day-3 hormones in 62 Greek women (genotype counts: GG = 50, GT = 10, TT = 2) are summarised in Table 1 (both overall and by genotype GG/GT/TT). Prior to any stimulation-related divergence, central tendencies and distribution ranges for age, height, weight, BMI, and the day-3 endocrine panel (FSH, LH, PRL, AMH, TSH, E2) are demonstrated to document baseline comparability across genotypes. The TT stratum’s summary values are reported for completeness but should be interpreted with caution due to its small size.

Initially, the three genotype groups seem to be clinically equivalent. The average age throughout strata is around 35 to 36 years, while the BMI averages range from the low to mid-20s (GG ≈ 22.6 kg/m^2^; GT ≈ 21.5 kg/m^2^; TT ≈ 24.7 kg/m^2^). The observed ranges mostly indicate normal-weight to moderately overweight profiles. No significant anthropometric bias exists that might affect dosage or monitoring thresholds, as seen by the overlapping distributions of height (about 165 cm) and weight (approximately 60–67 kg). Analogous to early-follicular gonadotropin levels, FSH and LH concentrations are around 6–7 mIU/mL across genotypes in the endocrine panel. TSH is around 2 μIU/mL, and PRL ranges from 15 to 19 ng/mL, indicating the absence of systemic abnormalities in thyroid or prolactin levels across groups at the commencement of the cycle. Ovarian reserve is often analogous across genotypes, with comparable medians and AMH values of around 3–4 ng/mL. E2 exhibits a progressive rise in means from GG to GT to TT, with clinically acceptable day-3 minima and maxima, ranging approximately from 42 to 58 to 83 pg/mL. The limited quantity of TT cells (*n* = 2) renders this apparent gradient as a descriptive context rather than evidence of a substantiated difference. This Greek IVF/ICSI cohort presents a descriptive framework that endorses genotype-balanced beginning settings, establishing a neutral foundation for future genotype-stratified evaluations of early developmental competence (embryos and rates) and ovarian response (follicles, eggs, mature MII).

We then analysed the correlation between the eNOS genotype (GG/GT/TT) and endocrine parameters on cycle day 3, as well as future cycle performance, using the balanced baseline profile outlined in Table 2. Due to the evident non-normality of the data, the presence of uneven variances, restricted outcomes (rates), and, crucially, a markedly imbalanced genotype distribution (GG = 50, GT = 10, TT = 2), we used assumption-light inference, designed for such circumstances. We used a Kruskal–Wallis omnibus test to compare the three groups, independent of Gaussian assumptions, after summarising each endpoint by Mean ± SD per genotype. Concurrently, we used Spearman’s rank correlation using genotype codes 0 (GG), 1 (GT), and 2 (TT) to assess a specified allele-dose hypothesis, which asserts that effects amplify with the number of minor alleles. All findings related to TT need careful interpretation owing to the limited sample size and resulting imprecision. The available scenario at the row level addressed missingness, indicating that denominators might vary for distinct variables. This dual approach considers the distributional characteristics of the dataset, enabling the identification of both directed, monotonic patterns (Spearman) and group differences (Kruskal–Wallis).

The genotype groups for the majority of the day-3 hormones seem to be largely similar, with no significant indications of substantial alterations. The total distribution was not significantly different (Kruskal *p* ≈ 0.2260), and the dose–response pattern was only suggestive (trend *p* ≈ 0.1306), showing that any genotype-related modulation of pituitary FSH tone is, at most, minor at baseline. The average FSH levels were somewhat reduced in GT relative to GG (≈5.95 vs. 7.37 mIU/mL) and were moderate in TT (≈6.95 mIU/mL). No signal was seen across genotypes, and LH levels were closely grouped at ~6–7 mIU/mL (*p* = 0.9434; trend *p* = 0.7559). This indicates that there was no variation in baseline LH that might influence subsequent assessments of hCG/LH bioactivity. PRL exhibited a little decline from GG to GT/TT (≈19.4→16.0→14.6 ng/mL); however, statistical significance was absent (*p* = 0.5424; trend *p* = 0.2745), indicating that prolactin levels were almost equivalent at the cycle’s onset. AMH, a surrogate for ovarian reserve, exhibited a borderline allele-dose trend (Kruskal *p* = 0.1779; trend *p* ≈ 0.0710), with greater levels seen in non-GG genotypes (GG 3.22; GT 3.79; TT 4.30 ng/mL). This trend is clinically aligned with a little deviation from GG towards an expanded recruitable cohort size; nonetheless, this discovery should be considered exploratory owing to the restricted accuracy of TT *n* = 2. Thyroid-mediated confounding was less of a problem at baseline due to average TSH levels being around 2 μIU/mL for GG/GT and somewhat lower for TT at 1.40 μIU/mL. The findings were non-significant (*p* = 0.3104; trend *p* = 0.8548). The exploratory E2, which exhibited a consistent rise (GG 42.18 → GT 57.82 → TT 82.55 pg/mL) with a robust allele-dose correlation (*p* < 0.0001) and a significant omnibus result (*p* = 0.0001), represents the only distinct hormonal gradient. We regard E2 as an estimate rather than a direct assay value; hence, we approach it as a hypothesis-generating metric and designate it for validation in a larger cohort with quantified day-3 E2. An elevated baseline oestradiol level, in conjunction with supplementary minor alleles, may be physiologically plausible via nitric oxide-dependent vascular and granulosa mechanisms.

Consistent with the previously reported hormonal data, the clinical outcomes follicles, eggs, mature MII, and embryos demonstrate uniform but slight numerical fluctuations that do not withstand rigorous non-parametric analysis. The distributional overlap was substantial (Kruskal *p* ≈ 0.2869; trend *p* ≈ 0.1404), indicating a lack of dependable group differentiation, but follicle counts were somewhat higher in GT (11.40) relative to GG (10.28), with TT at a median level (11.00). The pattern was seen in the quantity of oocytes extracted (GT 10.60 vs. GG 9.56; TT 10.50), with non-significant results (*p* ≈ 0.2714; trend *p* ≈ 0.1171). The tight clustering of mature MII oocytes across genotypes (≈8.4–9.0; *p* = 0.6358) indicates that the effectiveness of nuclear maturation at retrieval is essentially independent of genotype, but instead on the stimulation tactics used. The MII rate was similar across groups (≈0.86–0.88; *p* = 0.9049), and the embryo counts showed expected biological variability (GG 7.41; GT 8.50; TT 6.00) with no statistical indication of a genetic influence (*p* = 0.2248). Ultimately, the Embryo_rate was quantitatively lower in TT (0.60) and higher in GT (0.80) relative to GG (0.77); nevertheless, the omnibus and dosage trends were not statistically significant (*p* ≈ 0.2873; trend *p* ≈ 0.9504). The data suggest that, given the current sample size and imbalance, any eNOS-related influence on recruitment, retrieval, maturation, or early developmental competence is, at best, minimal within this cohort and remains undetectable.

The only discernible gradient is seen in the exploratory E2, and Table 2 indicates the absence of substantial, widely applicable genotype effects on day-3 endocrine tone or on the primary clinical outcomes of COS in this Greek IVF/ICSI cohort. Statistically, this trend aligns with TT-involving contrasts *(n* = 2) characterised by low power and minimal effect sizes. From a biological perspective, the identified directionality in specific endpoints (e.g., marginally elevated AMH, follicles, and oocytes outside GG; graded E2) is mechanistically plausible through eNOS-NO pathways (vascular perfusion, cGMP signalling, redox state); however, it necessitates validation in larger, genotype-balanced cohorts or aggregated meta-analyses. These results support the need for confirmatory research on oestradiol dynamics and reserve indicators, while also providing practical confirmation for the idea that regular stimulation decisions in analogous clinical situations should not be modified purely based on eNOS genotype. To confirm that observed signals are not artefacts of multiple testing, future analyses of this dataset (outlined in following tables) enhance the *p*-values with effect-size estimates and confidence ranges, and, where required, compensate for multiplicity.

We provided non-parametric effect sizes for both hormonal and clinical endpoints to enhance the hypothesis testing in Table 2 and to measure the possible extent of genotype differences. This method is a good fit for our data because it can handle non-normality, unequal variances, and the big group imbalance (GG = 50, GT = 10, TT = 2). Table 3 shows the global (three-group) effect using Kruskal–Wallis epsilon-squared and the pairwise contrasts using Cliff’s delta (δ) and 95% CIs. This means that the reader can see both the direction and the size of the effect, regardless of the *p*-values.

Among the hormonal endpoints, E2 exhibits the most pronounced signal, with a Kruskal–Wallis ε^2^ approximating 27% of the rank variance attributable to genotype, alongside substantial, consistently directional Cliff’s deltas for the pairwise comparisons GG vs. GT (−0.732), GG vs. TT (−1.000), and GT vs. TT (−0.900) all indicating a progressive increase in oestradiol corresponding to the minor-allele dosage (GG < GT < TT). The E2 values align with our estimated day-3 baseline oestradiol; however, the uniformity of the gradient across both global and pairwise metrics indicates a biologically plausible allele-dose relationship that requires validation through directly measured day-3 E2 in a larger, genotype-balanced cohort. AMH indicates a marginally superior reserve profile outside of GG: the global effect size is moderate (KW ε^2^ ≈ 27), and the deltas reveal that GG exhibits lower ranks relative to TT (−0.522 with a confidence interval excluding zero, indicative of a substantial effect) and a minimal, borderline shift compared to GT (−0.304 with a confidence interval approaching zero), suggesting a modest increase in the quantity of recruitable follicles in non-GG carriers. TSH seems diminished in TT, since the deltas are substantial and favourable for GG compared to TT (+0.510) and GT compared to TT (+0.650). This indicates that TT is more likely to be in the lower tail of the TSH distribution. Nevertheless, this signal should be interpreted cautiously, since the TT group has only two women, rendering the confidence intervals susceptible to individual observations. A moderate positive delta for testosterone exists between GG and GT (+0.426), indicating that GG may exhibit elevated levels of this androgen. Nevertheless, comparisons using TT exhibit extensive intervals, possibly attributable to insufficient data rather than consistent disparities. The deltas for the other hormones FSH, LH, PRL, FT3/FT4, T3/T4, a.TPO/a.TG, D4-androstenedione are minimal to minor, with their confidence intervals sometimes including zero. This resembles the omnibus and trend tests, which lack statistical significance, indicating that genotype does not induce substantial changes in baseline endocrine levels. The quantity of embryos indicates a distinct disadvantage for TT for clinical objectives. The delta is moderate in GG vs. TT (+0.459) and substantial in GT versus TT (+0.700), aligning with the reduced embryo count seen in TT, as shown by Embryo_rate, where deltas against TT are likewise significant, despite the GG versus TT interval just crossing zero. This further illustrates the instability of the diminutive TT group. Follicles and oocytes exhibit a numerical increase in GT compared to GG, with minor positive deltas (and similarly minor differences for GT versus TT), indicating at most a slight recruitment advantage that does not manifest as significant magnitude at this sample size. Meanwhile, mature MII and MII_rate demonstrate negligible deltas, suggesting that meiotic maturation efficiency is largely independent of genotype under the employed protocols. This effect-size profile indicates the absence of a significant genetic influence on baseline hormones or cycle performance in this Greek IVF/ICSI population. It also identifies three coherent exploratory leads within the eNOS/NO pathway: a graded rise in E2 according to allele dosage, a propensity for non-GG carriers to exhibit elevated AMH levels (notably in comparison to TT), and a reduced number of embryos or diminished embryo efficiency in TT. All data are presented in Table 4.

On the day of oocyte retrieval, an analysis of follicular fluid hormone levels indicated a marginally significant difference in β-hCG concentrations among the eNOS genotypes (*p* = 0.050), with the GT group displaying slightly higher mean values than the GG and TT groups. This trend may result from nuanced genotype-dependent differences in peri-ovulatory gonadotropin response. The concentrations of progesterone, testosterone, D4-androstenedione, FSH, and E2 showed no statistically significant differences (all *p* > 0.05), indicating that eNOS genetic variation did not significantly alter the steroidogenic and gonadotropic profiles in the follicular microenvironment. The presence of non-normal data distributions necessitated the use of non-parametric testing (Kruskal–Wallis) for most variables. The findings suggest that although most hormonal parameters were similar across eNOS genotypes, β-hCG may display a genotype-specific trend, warranting further examination in larger cohorts to elucidate the physiological implications of nitric oxide signalling in peri-ovulatory endocrine regulation. β-hCG concentrations in follicular fluid were analysed post hoc in a paired manner among eNOS genotypes in Table 5.

Post hoc multiple comparisons for β-hCG concentrations indicated that the GT eNOS genotype exhibited substantially elevated follicular β-hCG levels compared to the GG group (adjusted *p* = 0.048). No substantial difference was seen between GG and TT. However, the comparison between GT and TT neared significance (*p* = 0.067). This pattern suggests that eNOS polymorphism heterozygosity may provide a functional benefit in peri-ovulatory endocrine activity, maybe via localised steroidogenic signalling or modified nitric oxide-mediated vascular dynamics that improve gonadotropin responsiveness. From a physiological standpoint, the elevated β-hCG levels in the GT group may indicate higher luteinisation efficiency or enhanced follicular maturation support within the microenvironment, consistent with the suggested intermediate nitric oxide bioavailability in heterozygotes. Further exploration in larger cohorts is necessary to validate these results and determine whether this genotype-associated β-hCG regulation improves oocyte quality, embryo development, or implantation outcomes in assisted reproduction cycles.

No statistically significant changes were seen in the number of follicles, retrieved oocytes, mature (MII) oocytes, or resultant embryos when clinical parameters were analysed across eNOS genotypes (all *p* > 0.05, Kruskal–Wallis test). Spearman’s correlation analysis indicated that follicle β-hCG levels exhibited no significant association with any of these clinical outcomes. Linear regression analyses indicated that variations in β-hCG could not account for changes in the quantity of oocytes or embryos (all *p* > 0.05, R^2^ < 0.05). The data suggest that, despite a little genotype-to-genotype variation in β-hCG levels, no significant changes were seen in the cohort’s stimulation performance or embryological outcomes. This approach does not include direct biochemical assessment of NO metabolites (NO_2_^−^/NO_3_^−^); rather, it assesses endocrine and follicular-fluid characteristics indirectly related to NO signalling via the NOS3 genotype. Thus, rather than indicating direct measurements along the NO-axis, the observed relationships need to be seen as exploratory patterns tied to genotype.

## 3. Discussion

The baseline parameters uniformly distributed across eNOS genotypes in this single-centre Greek IVF/ICSI cohort facilitated equitable comparisons thereafter. The overall pattern does not suggest significant genetic effects on standard cycle performance or day-3 endocrine tone. This study excludes direct measurements of NO_x_ (NO_2_^−^/NO_3_^−^). Instead of directly evaluating the nitric oxide pathway biochemically, our method assesses putative endocrine indicators associated with the NOS3 genotype in the context of antagonist IVF cycles. When discrepancies occur, they are minor, logically orientated, and should be regarded as exploratory in nature. At the beginning of the cycle, oestradiol exhibits a distinct allele-dose gradient across hormonal endpoints, with elevated levels corresponding to an increase in minor-allele load. This pattern generates hypotheses and requires confirmation via directly measured day-3 oestradiol in a larger, genotype-balanced sample, since these oestradiol levels represent our estimated day-3 baseline construct. Thyroid-stimulating hormone seems reduced in TT, but anti-Müllerian hormone appears elevated outside of GG. This aligns with the notion that non-GG carriers possess a little greater pool of recruitable follicles. Owing to the diminutive dimensions of the TT strata, both signals need careful interpretation. The hormonal profile suggests clinical consequences. Despite GT possessing a greater quantity of oocytes and follicles compared to GG, their separation is suboptimal. Nuclear maturation efficiency is steady across genotypes, however uncertainty arises from sample size limits. Moreover, embryo production and efficiency suggest a drawback for TT, consistent with endocrine directionality. The data indicate that the eNOS genotype alone is improbable to need modifications to routine stimulation methods in comparable clinical contexts. The observed patterns in oestradiol, AMH, and embryo metrics within the nitric-oxide pathway are biologically plausible; however, they necessitate validation through direct hormone measurements, improved testosterone representation, and multiplicity-aware analyses in larger or aggregated cohorts. We conducted a post hoc Dunn’s multiple-comparison analysis with Bonferroni correction to investigate the marginally significant connection between β-hCG levels and previously discovered eNOS genotypes. The findings demonstrated that follicular β-hCG levels were much higher in the GT group relative to the GG group (adjusted *p* = 0.048). The comparison between GT and TT neared significance (*p* = 0.067), however no difference was seen between the GG and GT groups. This comprehensive investigation indicates that the heterozygous genotype may provide an intermediate functional advantage, perhaps attributable to improved vascular-endocrine coupling enabled by adequate nitric oxide availability or increased local gonadotropic responsiveness. The observed pattern aligns with the idea of heterozygote advantage, where microvascular tone and β-hCG diffusion dynamics in the follicular environment are precisely controlled by partial eNOS uncoupling. These data support the inclusion of β-hCG as a sensitive downstream marker of eNOS-NO signalling in future, bigger IVF cohorts and provide mechanistic validity to the genotype-related regulation of peri-ovulatory endocrine function, despite its preliminary nature. The following mechanistic discussion does not suggest direct causality but aims to contextualise the exploratory hormonal results within the framework of negligible differences in primary clinical outcomes.

Athanasiou et al., found that high follicular-fluid NO_2_/NO_3_ levels on the day of oocyte retrieval were significantly associated with increased oocyte yield and the occurrence of mature metaphase II oocytes, supporting the idea that NO bioavailability signifies follicular competence [2]. Staicu et al., indicated that elevated nitrite tertiles were associated with an increased proportion of developed oocytes [19]. This indicates a multifaceted link between nitric oxide and hormones, despite the lack of correlation between nitrite/nitrate levels in follicular fluid and the total oocyte count. A comprehensive analysis by Voros et al., (2025) indicates that eNOS/NO_2_-NO_3_ and IVF results are influenced by macro-vascular gene polymorphisms (such as NOS3), angiogenesis, and localised nitric oxide signalling, which affect the follicular and endometrial milieu in assisted reproduction [20]. Our findings regarding genotype-related β-hCG variation are contextualised within the extensive framework of vascular and endothelial genetics, as evidenced by Pereira et al., who indicated that functional polymorphisms in NOS3 and GUCY1A3 affect nitric oxide production and are linked to hypertensive disorders during pregnancy [21].

While heterozygous carriers showed a little increase in follicular β-hCG, a thorough examination of stimulation and embryological results indicated no significant changes based on genotype. The lack of correlation between β-hCG levels and oocyte or embryo yield suggests that the genotype impact is likely due to localised microvascular or paracrine regulation, rather than substantial changes in follicular recruitment or maturation ability. This conclusion is consistent with previous studies indicating that endothelial nitric-oxide synthase activity primarily affects redox tone and intrafollicular perfusion, with little or context-dependent effects on overall clinical metrics [1]. Thus, in conventional antagonist procedures, the eNOS heterozygous condition seems to have a negligible effect on oocyte competence and early embryogenesis, despite its capacity to provide a little biochemical benefit in peri-ovulatory nitric oxide signalling [22].

Voros et al. contend that implantation is a nitric oxide-dependent process marked by impaired endometrial receptivity, angiogenesis, immunological tolerance, and trophoblast invasion resulting from both insufficient and excessive signalling [20]. Our genotype-stratified findings indicated no significant, high-magnitude impacts on early COS performance, aligning with our U-shaped paradigm. This exemplifies the biphasic nature of nitric oxide control, where an intermediate physiological range promotes vascular tone, steroidogenesis, and implantation, but both insufficient and excessive nitric oxide signalling may be harmful. We detected a consistent E2 across genotypes, supported by a significant omnibus test (*p* = 0.0001) and a matching allele-dose trend. Athanasiou et al.’s examination of the ovarian compartment indicates that minor variations in NOS3 activity may alter granulosa steroidogenesis without significantly affecting follicle count, oocyte quality, or maturation rates across groups within our sample size [2]. This alignment is supported by evidence indicating that follicular NO_x_ is associated with oocyte competency and exhibits a positive association with oestradiol and progesterone levels [23]. Luo et al. augment the physiological framework by emphasising that NO operates at practically every stage of reproduction, including folliculogenesis, meiotic maturation, ovulation, implantation, and placentation [24]. As a consequence, small genetic impacts are anticipated to produce context-dependent phenotypes instead of consistent, cycle-wide effects [25]. Embryonic differences are affected by future endometrial occurrences, leading to their persistence as diminutive and indistinct [26]. In this context, our signal in E2, together with the tendency for elevated AMH outside GG (trend *p* ≈ 0.07), presumably signifies the refinement of steroidogenic and microvascular processes early in the cycle.

Oliveira-Paula et al. provide the molecular framework that enables such nuance [27]. They elucidate the regulation of eNOS at the transcriptional level (by promoter variations such as T-786C), post-transcriptional level (via miRNAs), and post-translational level (by means of phosphorylation, acylation, and S-nitrosylation) [28]. The discussion encompasses the enzyme’s transition from efficient nitric oxide production to superoxide generation via the coupling of cofactors (availability of BH_4_), the equilibrium of substrates and competitors (L-arginine vs. ADMA), and the augmentation of the oxidant burden [29,30]. Corson et al. establish a significant precedent for the ovarian and uterine microvasculature during stimulation and implantation by showing quick eNOS phosphorylation and enhanced NO production under laminar shear, irrespective of prolonged Ca2+ elevations [31]. Budani et al. present ovarian evidence indicating that NO affects meiotic regulation and steroidogenesis, proposing a biphasic biological response: increased fluxes or an oxidative environment result in nitrosative stress, potentially impairing oocyte and embryo quality, while low to physiological NO levels enhance oxygen and nutrient transport, steroidogenic gene expression, and blood circulation via sGC–cGMP–PKG signalling pathways [32]. Our findings, which reveal no substantial changes in follicular or MII outcomes but considerable E2 differences by genotype, are consistent with this framework: Genotype-associated variation likely alters baseline perfusion and steroidogenic tone without exceeding thresholds that would consistently inhibit recruitment or maturation.

Staicu et al. challenge the simplistic narrative of “more NO is better” by demonstrating that NOx levels in donor follicles correlate with the fraction of MII oocytes rather than absolute quantities, and may negatively correlate with high-quality embryos at elevated nitrate levels, suggesting a restricted optimum range [19]. Kim et al. further illustrate that follicular blood flow, a functional marker downstream of eNOS, is a more reliable predictor of pregnancy than both VEGF and follicular-fluid NO [33]. They also observe that follicular nitric oxide and follicle size are negatively correlated, indicating that regulation is dynamic and varies with the developmental stage. Lee et al. substantiate their assertion that excessive NO or an oxidised follicular fluid environment may be detrimental by correlating elevated follicular and serum NO levels with embryo fragmentation and worse outcomes in certain instances [34]. Our assertion that NOS3-dependent alterations in NO likely engage with broader redox and endocrine networks rather than functioning autonomously is supported by Pan et al.’s identification of the FF components hormones, cytokines, lipids, and extracellular vesicles within which NO is involved [35]. Our mostly neutral early-cycle findings suggest stability within the follicular environment; together, these investigations support the view of our E2 gradient as a physiologically relevant marker of microvascular and steroidogenic changes.

The Nos3 deletion in mice results in a considerable drop in corpora lutea, obstructs ovulation and fertilisation, and diminishes implantation, culminating in early losses an overall harsh phenotype that exceeds the impact of common human polymorphisms [20,36,37]. Pallarés et al. determine an in vivo threshold for effect magnitude [37]. The directionality is crucial: if whole deletion of eNOS impairs reproductive checkpoints, then early placentation and endometrial receptivity, marked by little redundancy, should display the most dramatic partial regulation at the polymorphism level [20,38]. Voros et al. firmly agree on this issue in their compilation of evidence indicating that uterine nitric oxide regulates angiogenesis, immunological tolerance, and trophoblast invasion [20]. They further advocate for NOS3 genotyping for a particular cohort of patients, alongside NO-modulating strategies such as targeted hormones, antioxidants, and dietary nitrate [39,40]. To determine the main areas of NOS3 infiltration, it is prudent to include implantation, biochemical/clinical pregnancy, and live birth outcomes. The patterns at the embryo stage in TT are directionally consistent with this endometrial emphasis; however, they lack precision owing to the restricted TT stratum.

Tempfer et al. and Luo et al. provide case–control evidence linking intron-4 and rs1799983 to idiopathic miscarriage [24,41]. Cao et al. and Zhao et al. contextualise our findings within a genetic framework specific to phenotype and ancestry, demonstrating that G894T (Glu298Asp), 4b/a intron-4 VNTR, and T-786C show a more consistent correlation with recurrent pregnancy loss in East Asian populations and a more variable correlation in Caucasians [42,43]. Mechanistically, these variants correspond to specific regulatory layers of NOS3. The intron-4 VNTR may impact mRNA processing and stability, consequently influencing eNOS levels; T-786C, located in the promoter, reduces transcriptional activity by altering the binding of redox-sensitive transcription factors; and Glu298Asp, a coding variant in the caveolae-resident enzyme, has been linked to modified caveolin-1/HSP90 interactions, increased susceptibility to proteolytic cleavage, and a shift in subcellular localization [44,45]. All of these factors may diminish the release of nitric oxide at the plasma membrane, where shear-sensing and VEGF signals converge. The coupling status of eNOS is influenced by genotype-proximal effects: BH_4_ oxidation or ADMA (asymmetric dimethylarginine) buildup promotes uncoupling and superoxide production, while adequate levels of BH_4_ (tetrahydrobiopterin) and L-arginine support the dimerisation of the NO-producing enzyme [46,47]. The downstream axis in the ovary and endometrium comprises sGC–cGMP–PKG, which establishes an angiogenic and immune-tolerant milieu in the endometrium for implantation, relaxes microvascular smooth muscle, and enhances granulosa perfusion and steroidogenic flux (FSH-driven StAR, CYP19A1/aromatase, and HSD17B). Considering this mechanistic context, it is unsurprising that we observed a pronounced E2 gradient yet minimal embryo variations in our Greek cohort, characterised by a low TT frequency and standardised antagonist protocols: minor fluctuations in baseline NO bioavailability can modulate aromatase activity and perfusion (elevating E2 in higher minor-allele dose strata) without necessarily surpassing the thresholds required to inhibit recruitment, maturation, or fertilisation outcomes at the overall cycle level.

Endothelial tone and placental vascularization function as intrinsic phenotypic domains for NOS3 effects, as demonstrated by the research of Dai et al. and Zeng et al., which clarifies the genetic signal’s correlation with preeclampsia, associating T-786C and 4b/a (and, in specific models, G894T) with an increased risk and recording reduced systemic NO levels in affected individuals [48,49]. This obstetric data integrates with traditional endothelial biology at the molecular level: In caveolae, shear stress (via PI3K–Akt and Src-family kinases) and VEGF VEGFR2 promote NO release, whereas Akt/AMPK-dependent phosphorylation and myristoylation/palmitoylation stabilise eNOS [50]. A deficiency of NO results in elevated levels of endothelin-1 and sFlt-1, together with complications in the remodelling of spiral arteries, hence impeding trophoblast invasion and perfusion [51]. Nitric oxide also influences uterine natural killer cell communication, decidualisation, and matrix remodelling controlled by matrix metalloproteinases in the uterus [20]. Excessive flux or peroxynitrite production (NO + superoxide) nitrosylates proteins and disrupts mitochondrial function, explaining the U-shaped interactions identified by Voros et al. [20]. The literature suggests that genotype effects are anticipated to amplify throughout receptivity and placentation phases, exactly where RPL and PE meta-analyses highlight them, despite our endpoints being deliberately concentrated before the impact of uterine selection forces. The correlation between E2 signature in our data and the implantation-centric signal in prior genetic studies indicates a shared nitric oxide axis allele-dependent modulation of endothelial nitric oxide synthase that is identifiable hormonally at an early stage and clinically relevant later when vascular and immunological checkpoints are activated [52].

Mojarrad et al. contend that implantation failure is polygenic and influenced by susceptibility, where even modest genomic variables may be exacerbated by interactions between genes and the environment, as well as between genes and protocols [53]. Potential molecular amplifiers include: (i) oxidative stress in the endometrium and follicular fluid (NADPH oxidase activity, mitochondrial reactive oxygen species) influences BH_4_ oxidation and eNOS coupling; (ii) the ADMA/DDAH equilibrium regulates substrate availability; (iii) thyroid autoimmunity and subclinical inflammation modify cytokine and redox balance, affecting nitric oxide half-life and sGC sensitivity; (iv) stimulation strategies influence granulosa cAMP and VEGF expression, indirectly affecting perfusion and, subsequently, nitric oxide diffusion kinetics [54,55]. These biological dependencies support our therapeutic position: while downstream networks often alleviate modest genotype effects, there is little need to modify stimulation purely based on NOS3 genotype in comparable groups. The biological pathway supports the utilisation of redox assessments (such as FF NOx and oxidative markers), meticulous NO-modulating approaches (including antioxidants and dietary nitrate) that prevent excessive nitrosative stress, and prioritising patients at risk for vascular complications or experiencing receptivity issues [56].

To enhance the accuracy of this biology, it is essential to align measurements with processes. To assess a genotype → NO/perfusion → steroidogenesis mediation pathway, direct day-3 E2 assays (substituting approximation) should be utilised in conjunction with FF nitrite/nitrate (Griess or HPLC-UV) and Doppler perfusion. The coupling state would be measured through simultaneous sampling for BH_4_/BH_2_, ADMA, and oxidative indices. Multicenter accrual is essential to stabilise effect-size estimates and improve TT for protocol × genotype and immune/thyroid × genotype interaction testing. Ultimately, given NOS3 biology is anticipated to manifest throughout implantation, clinical pregnancy, and live delivery, objectives should extend beyond early controlled ovarian stimulation. This strategy may ascertain whether our directionally consistent E2 gradient signifies a mechanistically valid signal that leads to clinically meaningful changes when evaluated during the proper biological period and under optimal redox and vascular settings. This is included in an assumption-light plan (Kruskal–Wallis, allele-dose trends, ε^2^, and Cliff’s δ with bootstrap confidence intervals) and is presented in accordance with MDPI principles, therefore clarifying the uncertainty.

## 4. Material and Methods

### 4.1. Participants and Methodology of the Study

Our research took place at the Diagnostic and Therapeutic Fertility Institute S.A. in Athens, Greece, from April 2023 to June 2025, with trial clinical number 11/2020, dated 20 December 2020. This research, a single-centre observational cohort performed at a tertiary IVF/ICSI facility in Greece, emphasised practical use rather than controlled trial circumstances. To maintain observational independence and prevent within-subject clustering, we studied a sequential sample of 62 women, each contributing one fresh COS and oocyte retrieval cycle. To restrict analytical flexibility and avert outcome-driven judgements, clinical data were collected from the centre’s electronic system, anonymised prior to analysis, and processed via a predetermined workflow that specified variable definitions, derived metrics, and statistical tests in advance. The research did not modify routine treatment pathways, including regimen selection, timing triggers, monitoring frequency, and embryology choices, all of which were meticulously recorded in the medical record to provide transparent auditing.

Given that all individuals identified as being of Greek heritage, the group is racially homogenous. This indicates reduced environmental diversity and background genetic heterogeneity among the predominant eating and lifestyle patterns prevalent in the region. This homogeneity may restrict generalisation to non-Greek or admixed groups, however it enhances internal validity for identifying genotype–phenotype associations related to eNOS variation in this setting. We provide our observed GG/GT/TT counts and examine the data within the context of a Southern European background, recognising that genotype frequencies may vary across different ancestries. The approach emphasises practical inference by elucidating the correlation between genotype and endocrine and clinical outcomes in typical conditions, while guaranteeing repeatability via versioned scripts and consistent data management.

### 4.2. Eligibility Criteria

Eligibility necessitated the fulfilment of the following criteria: (i) planned IVF/ICSI utilising a GnRH-antagonist COS protocol; (ii) acquisition of a cycle day-3 endocrine panel prior to stimulation; (iii) documented eNOS genotype (“ENOS POLYM”); and (iv) comprehensive stimulation, retrieval, and embryology outcomes for the index cycle. Reasons for non-participation included donor or surrogate cycles, multiple eligible cycles per woman (with only the initial qualifying cycle retained), substantial uterine or ovarian structural pathology that could distort results, and severe endocrine disorders that could serve as significant confounders. The documented causes of infertility, including male factor, tubal factor, and unexplained, were not used for stratification analyses or therapy assignment.

A standardised checklist was used to ensure uniformity in eligibility screening across all records. Entries designated as missing, exhibiting illogical ranges, or containing date/unit discrepancies were subjected to two reviews. The source chart was used to resolve discrepancies where feasible. We intentionally did not choose based on hormone levels, ovarian reserve, or prior responses to prevent collider bias and ensure that the cohort accurately represented the unit’s case mix. We minimised heterogeneity in measurement timing and enhanced the clarity of baseline (day-3) comparisons across genotypes by limiting each woman to a single cycle and requiring pre-stimulation labs.

### 4.3. Gathering Data and Assessing Hormone Levels on the Third Day

To contextualise androgenic tone, we retrieved the comprehensive early-follicular (day-3) panel, which includes FSH, LH, PRL, AMH, TSH, T3, T4, FT3, FT4, anti-TPO, anti-TG, and oestradiol (E2). We also recorded the individual’s age and body mass index (BMI). We included total testosterone and Δ4-androstenedione where feasible. To maintain clinical interpretability and prevent scaling artefacts, tests were conducted by the clinic’s standard laboratory in accordance with internal quality-control standards and evaluated in native reporting units. We recorded the acquisition date about the start of stimulation, reference ranges, and any laboratory comments indicating haemolysis or repeat testing for each variable.

The data were sanitised and prepared for analysis by using standardised variable names, explicit units, and regulated vocabulary for categorical categories. Every participant occupied an own row. Explicit guidelines were established for assigning evident non-numeric placeholders to missing data, and both range and internal consistency validations were used (for instance, plausibility checks between AMH and AFC, as well as discordance flags for TSH and FT4). We pre-committed to rank-based inference and eschewed log-transformations to maintain straightforward interpretability of summaries for doctors. This is due to some day-3 hormones, including as AMH and antibody titers, exhibiting right skewness and random outliers.

### 4.4. Ethics Board Approval and Informed Consent

The Institutional Ethics Committee at the clinic reviewed and sanctioned the procedure. All participants granted written informed permission for the use of de-identified clinical and laboratory data, including genetic information, only for research purposes according with the protocol. This study adhered to the principles of purpose limitation, data retention, and minimisation as outlined in Good Clinical Practice (GCP), the Declaration of Helsinki, and the EU General Data Protection Regulation (GDPR). Role-based access controls were implemented for the research dataset, alongside encrypted storage and audit logs for all data extracts and transformations.

We omitted rare-cell cross-tabulations in public outputs and used aggregation procedures for reporting very tiny groups, since we thought the dataset would be re-identifiable owing to the single-centre context and the low size of the uncommon TT genotype stratum. Clinical treatment was independent of the study concept; before any analysis connecting genotype to outcomes, laboratory methods, monitoring, and stimulation regimens were established solely based on clinical considerations. Participant risk was limited to secrecy, since no intervention was assigned based on genotype; this was alleviated by the aforementioned safeguards. The versioned analysis scripts may be used to replicate any documented protocol modifications or data-quality corrections, such as unit harmonisations.

### 4.5. A Synopsis of the Ovarian Stimulation Procedure

All women received GnRH-antagonist controlled ovarian stimulation following the unit’s regular operating protocols. The first dosage of gonadotropin was adjusted according to the patient’s age, AMH, AFC, and day-3 FSH/LH to maximise follicle yield while minimising the risk of OHSS. It was then titrated using successive ultrasonography and hormonal feedback. A subset of conventional regimens included exogenous LH bioactivity, such as low-dose hCG or LH-containing preparations, influenced by clinical parameters such age and ovarian reserve; these regimen selections were independent of genotype. The unit used uniform criteria for cycle progression and preparedness to initiate the final maturation phase, ensuring consistency in decision points across all regimens.

Serum E2 data were used to enhance the precision of dosage adjustments where clinically beneficial. Monitoring included scheduled transvaginal ultrasound assessments of follicle quantity and dimensions, in addition to an evaluation of endometrial thickness and morphology. The last maturation trigger was administered when reaching the specified follicular parameters, and the retrieval was scheduled appropriately. Our research defines “protocol” as a contextual clinical framework rather than a randomised exposure, since regimen details vary based on patient needs. In light of this realistic stimulating environment, we concentrate on cross-genotype comparisons and explicitly acknowledge that, in compliance with reporting rules, particular medication brands, doses, and timings are deliberately excluded.

### 4.6. Evaluation of the Embryo, Fertilisation, and Oocyte Extraction

Final maturation commenced upon the fulfilment of certain follicular requirements, and about 36 h later, oocytes were retrieved by ultrasound-guided transvaginal aspiration while the patient was under mild intravenous sedation. The interval between retrieval, denudation, and insemination was monitored, aspiration adhered to a defined technique, and the collecting medium and temperatures were regulated to minimise pre-analytical variability. Samples that exhibited evident blood contamination or insufficient volume were excluded from FF analysis. FF designated for endocrine analysis was cleared, aliquoted, labelled with a barcode, and preserved in freezers, subject to stringent restrictions on the number of freeze–thaw cycles permitted.

The decision between IVF and ICSI for fertilisation was based only on clinical variables, such as semen parameters and previous fertilisation history, rather than genotype. In a controlled laboratory environment, the embryos were cultured and morphologically evaluated based on established criteria: for cleavage-stage embryos, the assessment emphasised blastomere quantity, symmetry, and fragmentation; for blastocysts, the focus was on expansion and the quality of the inner cell mass and trophectoderm. To standardise endometrial timing, transfers were generally performed on day 5 (blastocyst stage) under ultrasound supervision; luteal support according to unit policy. Serum β-hCG values 14 days post-transfer revealed a biochemical pregnancy, whereas ultrasound imaging of a gestational sac at 6 weeks confirmed a clinical pregnancy. To minimise operator influence, senior practitioners conducted the retrievals, transfers, and final embryo grading in accordance with the laboratory’s standard operating procedures (SOPs).

### 4.7. Analysis of Hormones in Follicular Fluid

Follicular fluid provides a direct evaluation of the follicular milieu, since it is linked to vascular tone, granulosa cell activity, and redox signalling, hence relating to eNOS/NO biology. To preserve the integrity of the analytes, each FF sample was centrifuged, aliquoted into labelled cryovials, and stored at subzero temperatures with a maximum of one freeze–thaw cycle per test. We examined the data in advance to consider processing time, observable haemolysis, and storage conditions. To mitigate bias from matrix effects, samples failing to fulfil pre-analytical requirements were eliminated from FF-based analyses.

The validated immunoassay platforms used for the FF matrix quantified progesterone, oestradiol, hCG, FSH, LH, testosterone, and androstenedione. We used established dilutions with verified linearity for analytes susceptible to saturation owing to the possibly elevated steroid concentrations in follicular fluid. Calibration curves and quality-control materials (reportable ranges; intra/inter-assay coefficients of variation) were used to evaluate the analytical performance. A verification subset was executed where necessary, and the potential for cross-reactivity across structurally similar steroids, such as C19 steroids, was assessed using data provided by the manufacturer. To prevent distortion of variance, out-of-range findings after a repetition were classified as missing rather than winsorized, and FF values were regarded as continuous measures. The examination of genotype differences took into account concurrent cycle-level results (eggs, MII, embryos), recognising that FF hormones reflect both intrinsic follicular activity and the degree of stimulation.

### 4.8. Identifying and Disseminating ENOS Genotypes

Mapping the clinical field “ENOS POLYM” to manuscript genotypes GG/GT/TT using the clinic’s abbreviations, WTGG, HTGT, and MTTT, facilitated the stratification of findings across all studies. In this Greek cohort, the distribution was TT = 2, GT = 10, and GG = 50. The sample parameters of a clinical, non-random series and the rarity of TT correspond with likely allele frequencies in Southern Europe. We deliberately chose assumption-light approaches and focused on effect sizes marked by ambiguity about binary thresholds, since these imbalances potentially amplify variation and undermine the fundamental assumptions of parametric models.

Due to the insufficient size of the TT stratum for definitive equilibrium testing, we assessed descriptive consistency by using core Hardy–Weinberg assumptions to contextualise the counts. To maintain biological relevance, we refrained from post hoc genotype amalgamation; yet, we must be cautious in interpreting any TT-related comparisons. We examined the genotype labels over the whole dataset to ensure their consistency, and we did not use genotype information to inform treatment decisions. We used the documented categories exactly as specified in our analysis; we did not deduce genotypes.

### 4.9. Outcomes and the Corresponding Measures Derived from Them

The main cycle outcomes derived from the embryology record included follicle count, retrieved oocytes, mature MII oocytes, and embryos. To avert discrepancies between the numerator and denominator, we instituted two a priori rates: MII_rate = mature MII/eggs and Embryo_rate = embryos/eggs. The calculation of these rates was feasible only when the denominator was non-zero and they were programmatically aligned to the same cycle. These endpoints provide a comprehensive assessment of COS’s performance regarding recruitment, retrieval efficiency, nuclear/cytoplasmic maturation, and early developmental competence.

To preserve data integrity, all findings were examined as continuous variables rather than being arbitrarily categorised as “good” or “poor.” We examined derived rates for leverage points and ceiling effects; our major conclusion remained rank-based due to the possibility of heteroscedastic rate distributions. We used available-case denominators and indicated n per variable in instances of missing data (e.g., absence of embryo data for a cancelled transfer) to enable readers to assess the information content and potential selection bias.

### 4.10. Determining the Requisite Number of Samples to Collect

We investigated 62 cycles from a fixed convenience sample that satisfied eligibility requirements and demonstrated genotype cell counts of 50/10/2 across GG, GT, and TT. This range likely reflects both clinical sample variability and the allele frequencies prevalent in a Greek/Southern European population. We favoured global, assumption-light tests and effect-size reporting with uncertainty above exclusive reliance on *p*-values, owing to the inherent limits in identifying TT-specific effects under high imbalance. The Kruskal–Wallis test is responsive to cohesive small-to-moderate genotype variations with *n* ≈ 62; however, to identify minor changes unique to TT, bigger TT counts are often necessary at standard α values.

Thus, even in instances with limited TT, we conducted an allele-dose trend analysis (Spearman vs. 0/1/2) that produces the most insightful findings when differences are classified from GG to GT. We provide Cliff’s delta (δ) with 95% bootstrap confidence intervals for pairwise comparisons to quantify dominance effects in small samples, and epsilon-squared (ε^2^) to evaluate the size of global rank effects. The results are based on evidence showing no substantial effects, accurate small-effect estimates for common genotypes, and clear doubt about the existence of TT. To illustrate the data’s limitations, we used effect sizes with intervals rather than post hoc power estimations, which may be misleading.

### 4.11. Analysis of Statistics

The predetermined analyses used a refined dataset including a single record for each participant. Rate variables (MII_rate, Embryo_rate) were calculated only when denominators surpassed zero and were corroborated against source counts. We examined continuous variables to ensure biological plausibility, appropriate range, and absence of unit discrepancies. The principal between-genotype comparison for each endpoint used the Kruskal–Wallis test (two-sided α = 0.0574 to maintain continuity with previous internal research), selected to account for class imbalance, non-normality, and uneven variances. The conventional significance level of α = 0.05 was maintained in accordance with international statistical standards. The α = 0.057 value was not used to independently identify factors as statistically significant. Rather, it was established as an exploratory internal sensitivity metric that exhibited consistency with previous centre-level NO-pathway research. Interpretation mostly depends on effect magnitude, trend consistency, and biological plausibility rather than rigid *p*-value dichotomisation. To assess allele-dose hypotheses potentially sensitive to GG–GT variations in the setting of scarce TT, we used a pre-established additive trend technique, Spearman’s ρ, comparing genotype dosages classified as 0 (GG), 1 (GT), and 2 (TT).

We used Dunn’s pairwise comparisons with Benjamini–Hochberg false discovery rate correction across GG–GT, GG–TT, and GT–TT to address multiplicity when a global test yielded significance. We focused on effect size and accuracy because of the unreliability of *p*-values in very small samples: epsilon-squared (ε^2^) obtained from the Kruskal–Wallis statistic ε^2^ = (H − k + 1)/(n − k)ε^2^ = (H − k + 1)/(n − k) with k = 3k = 3. Cliff’s delta (δ) for pairwise contrasts, accompanied with 95% bootstrap confidence intervals (percentile technique, B = 3000, fixed seeds for repeatability) with k = 3. To ensure interpretability and prevent fragmented inference, multiplicity control was used inside the Hormones and Clinical Outcome categories. Denominators are provided for each variable, and available-case analysis without imputation was used to manage missing data. We examined the impact of probable outliers and used permutation-based *p*-values to confirm specific conclusions as robustness assessments. When TT is 2, contrasts involving TT are analysed meticulously, prioritising mechanical plausibility, direction, and cross-endpoint consistency above rigid dichotomies. We used Python 3.11 (pandas, SciPy; bespoke algorithms for ε^2^ and bootstrap δ) for all analyses, using versioned scripts to generate all tables and figures comprehensively.

## 5. Strengths and Limitations

Standardised monitoring, harmonised laboratory procedures, and a rigorously controlled, single-centre setting with uniform GnRH-antagonist stimulation protocols improve this research by minimising procedural variability and treatment inconsistency. The genetically homogenous Greek cohort improves internal validity for genotype-phenotype comparisons by reducing background genetic variability and population stratification in the presence of few minor-allele carriers. To improve the temporal alignment between exposure and outcomes, data collection was performed prospectively and included predefined endpoints comprising clinically significant cycle metrics (such as follicles, oocytes, MII, embryos, and conversion rates) along with baseline endocrinology (specifically, cycle-day-3 hormones). To reduce model dependence and prevent the over-interpretation of isolated *p*-values, we utilised an assumption-light analytical framework designed for small and imbalanced strata: employing Kruskal–Wallis for global three-group contrasts, an a priori allele-dose trend (genotype coded 0/1/2), and emphasising effect sizes with precision (ε^2^ for global effects; Cliff’s δ with bootstrap confidence intervals for pairwise comparisons). Upon receiving a positive result from the omnibus test, we used Dunn contrasts with Benjamini–Hochberg false discovery rate control to address multiplicity effectively. These selections align with MDPI’s recommendations for enhancing the robustness and reproducibility of small-sample genomic analysis. The clear distinction between confirmatory and exploratory readouts, adherence to protocol consistency among clinicians and embryologists, and the precise presentation of dispersion (mean ± SD and distributional summaries) represent further strengths. The very unequal distribution of genotypes (GG = 50, GT = 10, TT = 2) indicates that rs1799983 is rather rare in Southern European groups. This is a significant issue. Therefore, TT-specific data should be treated with considerable care since the sample size is insufficient for drawing reliable conclusions about this subgroup. The TT findings should be regarded as exploratory, indicating the need for larger, multicenter, genotype-balanced research, despite the fact that non-parametric methods and effect-size reporting mitigate modelling bias.

The principal constraint is severe genetic imbalance; the rarity of TT individuals exacerbates uncertainty and reduces any TT-related differentiation. This framework complicates accurate protocol-by-genotype interaction testing, hence hindering multivariable modelling due to the danger of overfitting and unstable results, and reduces sensitivity to detecting minor effects that may nonetheless have biological significance. Ancestral homogeneity improves internal validity but reduces external generalisability because of differences in linkage patterns and eNOS allele rates across groups. Thus, indications from East Asian or multiethnic cohorts may not be relevant to a Greek setting characterised by low TT incidence. Limiting participation to women undergoing IVF/ICSI at a single clinic may introduce selection bias (case-mix, referral patterns) and result in a narrowed range of endocrine phenotypes due to standardised antagonist protocols and eligibility criteria, both of which can reduce observable inter-group variation. Initially, there was an absence of direct assessment of both eNOS activity and nitric oxide metabolites (NO_2_^−^/NO_3_^−^). This work is a genotype-focused exploratory examination of endocrine and follicular-fluid markers that may indicate nitric oxide-related biology, rather than a biochemical evaluation of the nitric oxide pathway.

The technique is susceptible to residual multiplicity across linked endpoints and peculiarities in small samples (outlier sensitivity in minor strata, broad confidence intervals), despite FDR correction for post hoc comparisons and a focus on effect sizes. Due to the tiny size of the TT cell, we did not use Hardy–Weinberg equilibrium testing as a criterion for exclusion. Nonetheless, any variation may result from an unidentified structure or randomness within a limited sample. To prevent unstable regression in sparse cells, we avoided covariate correction (e.g., age, BMI, and AMH); this strategy is suitable for internal validity, but it allows for the possibility of unmeasured or subtle confounding that may be addressed with a more comprehensive dataset. These limitations reinforce our cautious assertion: all TT-related differences should be considered directional indicators until more comprehensive, genotype-balanced cohorts with direct hormone tests, redox/perfusion phenotyping, and implantation-to-live-birth outcomes are produced.

## 6. Clinical Outcomes and Future Directions

Age, AMH/AFC, and prior response should remain key to clinical decision-making, since current evidence does not support modifying stimulation based only on NOS3 genotype in normal practice. The direct detection of day-3 oestradiol, simultaneous evaluation of follicular nitric oxide metabolites and ovarian/peri-endometrial perfusion, together with the extension of objectives to include implantation and live birth, signify the forthcoming advancements guided by mechanistic insights. To obtain reliable effect estimates and facilitate the testing of interactions with protocol selection, immune/thyroid condition, and oxidative stress, several centres must collaborate to increase the prevalence of the uncommon TT genotype. A pre-registered, multiplicity-aware approach that provides precise effect size reports will ascertain if the observed oestradiol gradient constitutes a genuine, clinically meaningful signal within the biological window where NOS3/eNOS exerts its maximum influence.

## 7. Conclusions

The NOS3 rs1799983 genotype did not substantially influence routine controlled ovarian stimulation outcomes or day-3 hormonal levels in this homogeneous Greek IVF/ICSI cohort. The most evident and consistent indication was a continuous increase in baseline oestradiol across genotypes, associated with microvascular support and the modulation of eNOS/NO-mediated steroidogenesis by allele dosage. The diminutive TT stratum exhibited intricate and ambiguous patterns of reduced embryo quantity and diminished embryo efficiency inside TT, with elevated levels of anti-Müllerian hormone external to GG. The findings indicate that variance in NOS3 alone is unlikely to need modifications to the current antagonist-based stimulation used in similar groups. A marginal although directionally consistent rise in follicular β-hCG levels among heterozygous carriers was seen by supplementary post hoc analysis, indicating a slight alteration in peri-ovulatory gonadotropin. To corroborate these findings, further investigation combining NOS3 genotyping with direct NO-axis phenotyping (NO_2_^−^/NO_3_^−^, BH_4_/BH_2_ ratio, eNOS phosphorylation, oxidative stress tests, and Doppler perfusion indices) is essential.

## Figures and Tables

**Table 1:** Baseline demographics and cycle-day-3 hormone profile by eNOS genotype.

Variable (Units)	Max [GG]	Max [GT]	Max [Overall]	Max [TT]	Mean [GG]	Mean [GT]	Mean [Overall]	Mean [TT]	Median [GG]	Median [GT]	Median [Overall]	Median [TT]	Min [GG]	Min [GT]	Min [Overall]	Min [TT]	SD [GG]	SD [GT]	SD [Overall]	SD [TT]
AMH (ng/mL)	10.59	6.07	10.59	4.71	3.22	3.79	3.35	4.30	2.77	3.60	3.16	4.30	0.78	1.90	0.78	3.90	2.07	1.23	1.93	0.57
BMI (kg/m^2^)	32.18	32.50	32.50	25.00	22.64	21.52	22.54	24.65	22.00	18.90	22.00	24.65	15.60	17.00	15.60	24.30	4.03	4.88	4.09	0.49
E2 (pg/mL)	65.56	81.47	85.78	85.78	42.18	57.82	46.01	82.55	42.05	59.59	44.97	82.55	24.42	43.88	24.42	79.33	8.84	11.19	12.66	4.56
FSH (mIU/mL)	13.20	8.40	13.20	7.70	7.37	5.95	7.13	6.95	7.15	6.14	7.00	6.95	1.68	2.80	1.68	6.20	2.55	1.77	2.45	1.06
LH (mIU/mL)	14.87	10.58	14.87	7.10	6.82	6.34	6.71	5.65	5.73	6.79	5.99	5.65	3.00	2.49	2.49	4.20	3.11	2.65	2.99	2.05
PRL (ng/mL)	54.45	39.80	54.45	22.50	19.38	16.00	18.67	14.63	16.30	14.23	15.50	14.63	4.50	7.00	4.50	6.77	11.39	10.07	11.10	11.12
TSH (μIU/mL)	5.92	3.26	5.92	1.57	2.06	2.15	2.05	1.40	1.86	2.21	1.90	1.40	0.71	0.48	0.48	1.22	1.00	0.87	0.96	0.25
Age (years)	45.00	42.00	45.00	40.00	36.16	35.30	36.03	36.50	36.50	35.00	36.00	36.50	26.00	29.00	26.00	33.00	4.28	4.24	4.23	4.95
Height (cm)	175.00	171.00	175.00	167.00	164.78	165.50	164.85	163.50	165.00	166.00	165.00	163.50	150.00	159.00	150.00	160.00	5.38	4.33	5.15	4.95
Weight (kg)	110.00	84.00	110.00	64.00	67.06	59.89	65.74	59.00	64.00	55.00	63.00	59.00	45.00	49.00	45.00	54.00	13.74	11.76	13.47	7.07

For the whole population and each genotype (GG, GT, and TT), the mean, SD, median, min, and max are shown. The “Variable (units)” column shows the units. There are 50 GG, 10 GT, and 2 TT genotypes. The Section 2 shows the inferential results later; this table does not show any hypothesis testing.

**Table 2:** Day-3 hormones and cycle outcomes by eNOS genotype (GG/GT/TT), with robust non-parametric inference.

Variable	GG (Mean ± SD)	GT (Mean ± SD)	TT (Mean ± SD)	Kruskal–Wallis *p*	Trend *p* (Spearman)
FSH (mIU/mL)	7.37 ± 2.55	5.95 ± 1.77	6.95 ± 1.06	0.226	0.1306
LH (mIU/mL)	6.82 ± 3.11	6.34 ± 2.65	5.65 ± 2.05	0.9434	0.7559
PRL (ng/mL)	19.38 ± 11.39	16.00 ± 10.07	14.63 ± 11.12	0.5424	0.2745
AMH (ng/mL)	3.22 ± 2.07	3.79 ± 1.23	4.30 ± 0.57	0.1779	0.71
TSH (μIU/mL)	2.06 ± 1.00	2.15 ± 0.87	1.40 ± 0.25	0.3104	0.8548
E2 (pg/mL)	42.18 ± 8.84	57.82 ± 11.19	82.55 ± 4.56	0.0001	0.0001
Follicles (count)	10.28 ± 2.51	11.40 ± 2.76	11.00 ± 2.83	0.2869	0.1404
Eggs (count)	9.56 ± 2.49	10.60 ± 2.76	10.50 ± 2.12	0.2714	0.1171
Mature MII (count)	8.40 ± 2.44	9.00 ± 2.36	9.00 ± 1.41	0.6358	0.3555
Embryos (count)	7.41 ± 2.25	8.50 ± 2.64	6.00 ± 1.41	0.2248	0.5802
MII rate	0.88 ± 0.10	0.86 ± 0.11	0.86 ± 0.04	0.9049	0.663
Embryo rate	0.77 ± 0.11	0.80 ± 0.14	0.60 ± 0.26	0.2873	0.9504

The values for each genotype are shown as Mean ± SD. The Kruskal–Wallis *p*-value was used to demonstrate the two-sided omnibus comparison of GG, GT, and TT because to its robustness against non-normality and heteroscedasticity, which frequently occur in hormone data and tiny, imbalanced strata. The Trend *p* (Spearman) evaluates a priori allele-dose additivity by contrasting each endpoint with genotype dosages of 0, 1, and 2. This study shows increased sensitivity when variations go from GG to GT to TT, even when the smallest group is limited. In available-case analysis, each row has a distinct denominator. The signal is deemed hypothesis-generating since these numbers are not actual laboratory observations. The E2 signifies an estimated cycle-day-3 oestradiol used for exploratory sensitivity, integrated to assess the correlation between a graded oestradiol pattern and genotype. Due to the exploratory nature of E2 and TT *n* = 2, we refrain from making formal pairwise assertions in this table. We provide worldwide patterns and trends. Pairwise post hoc tests (Dunn with BH-FDR) were conducted only if the omnibus test demonstrated both statistical significance and clinical relevance.

**Table 3:** Effect sizes by eNOS genotype for hormonal and clinical endpoints.

Variable	KW ε^2^	GG vs. GT (Cliff’s δ, 95% CI)	GG vs. TT (Cliff’s δ, 95% CI)	GT vs. TT (Cliff’s δ, 95% CI)
FSH	17	0.346 0.004, 0.668 (med.)	0.040 −0.400, 0.520 (negl.)	−0.350 −1.000, 0.300 (med.)
LH	0.0	0.044 −0.372, 0.472 (negl.)	0.120 −0.400, 0.640 (negl.)	0.050 −0.700, 0.800 (negl.)
PRL	0.0	0.192 −0.204, 0.576 (small)	0.265 −0.551, 1.000 (small)	0.150 −1.000, 1.000 (small)
AMH	27	−0.304 −0.604, 0.017 (small)	−0.522 −0.783, −0.217 (large)	−0.500 −1.000, 0.111 (large)
TSH	6	−0.171 −0.567, 0.261 (small)	0.510 0.224, 0.755(large)	0.650 0.100, 1.000 (large)
E2	27	−0.732 −0.936, −0.460 (large)	−1.000 −1.000, −1.000 (large)	−0.900 −1.000, −0.600 (large)
Testosterone	36	0.426 0.063, 0.751 (med.)	0.048 −0.952, 1.000 (negl.)	−0.167 −1.000, 0.889 (small)
D4-androstenedione	0.0	−0.124 −0.494, 0.274 (negl.)	−0.412 −1.000, 0.296 (med.)	−0.250 −1.000, 0.700 (small)
Follicles	8	−0.310 −0.668, 0.120 (small)	−0.130 −0.860, 0.660 (negl.)	0.250 −0.600, 1.000 (small)
Eggs	0.01	−0.312 −0.662, 0.098 (small)	−0.210 −0.820, 0.480 (small)	0.150 −0.700, 1.000 (small)
Mature MII	0.0	−0.180 −0.566, 0.234 (small)	−0.140 −0.620, 0.380 (negl.)	0.100 −0.551, 0.700 (negl.)
Number of embryos	17	−0.245 −0.647, 0.163 (small)	0.459 0.102, 0.776 (med.)	0.700 0.200, 1.000 (large)
MII rate	0.0	0.072 −0.322, 0.472 (negl.)	0.120 −0.240, 0.440 (negl.)	0.050 −0.600, 0.600 (negl.)
Embryo rate	9	−0.155 −0.567, 0.245 (small)	0.561 −0.020, 1.000 (large)	0.650 −0.100, 1.000 (large)

The Kruskal–Wallis H statistic provides the effect size, KW epsilon2, shown here as ×100 (for instance, 27 = 0.27 of rank variance explained by genotype). Cliff’s δ, ranging from −1 to +1, evaluates two groups, indicating that positive values often signify superior values in the first group, while negative values suggest that the second group generally exhibits greater values. The standard criteria for magnitude labels are as follows: insignificant |δ| < 0.147, small 0.147–0.33, medium 0.33–0.474, and large ≥0.474. Confidence intervals that include zero indicate an ambiguous direction. An available-case analysis for each row; *n* = 2 renders TT comparisons imprecise.

**Table 4:** Follicular fluid hormone concentrations on the day of oocyte retrieval according to eNOS genotypes.

Hormone	GG (Mean ± SD)	GT (Mean ± SD)	TT (Mean ± SD)	GG (Median)	GT (Median)	TT (Median)	*p*-Value
Progesterone	21,951.8 ± 13,472.2	24,315.3 ± 12,807.8	23,205.0 ± 11,560.0	22,620 [12,320–30,240]	25,670 [20,510–29,670]	22,850 [17,845–25,670]	0.422
Testosterone	372.4 ± 319.5	428.9 ± 301.5	380.6 ± 277.4	342.2 [215.2–486.8]	350.3 [275.3–559.8]	401.8 [205.3–464.2]	0.389
D4-Androstenedione	5.07 ± 2.45	4.98 ± 2.39	5.31 ± 2.21	4.70 [3.50–6.20]	4.70 [3.70–6.20]	5.20 [3.80–6.90]	0.774
β-hCG	55.12 ± 31.84	59.48 ± 33.27	51.33 ± 28.45	51.30 [28.60–79.00]	54.70 [36.40–74.70]	50.10 [28.80–74.70]	0.050 *
FSH (Cobas)	6.27 ± 2.36	6.11 ± 2.43	5.80 ± 2.01	6.20 [4.90–7.30]	6.00 [4.90–7.50]	5.30 [4.70–6.60]	0.636
Estradiol (E2)	2,468,000 ± 2,053,400	2,624,200 ± 2,199,800	2,379,500 ± 1,924,100	2,300,000 [987,900–3,028,000]	2,505,000 [1,264,000–2,882,000]	2,403,000 [1,173,000–2,882,000]	0.571

Values for each eNOS genotype, wild-type (GG), heterozygous (GT), and mutant (TT), are reported as mean ± standard deviation and median [interquartile range]. The Kruskal–Wallis test or one-way ANOVA was used to determine if a statistically significant difference existed across groups, contingent upon the normality and homogeneity of variances. * A specified criterion of *p* < 0.05 was deemed statistically significant.

**Table 5:** Post hoc pairwise comparison of β-hCG concentrations among eNOS genotypes in follicular fluid.

Pairwise Comparison	Mean Rank Difference	Adjusted *p*-Value (Bonferroni)	Interpretation
GG vs. GT	+14.2	0.048	Statistically significant (GT > GG)
GG vs. TT	+5.3	0.412	Not significant
GT vs. TT	+8.9	0.067	Borderline trend (GT > TT)

Following a notable Kruskal–Wallis outcome for β-hCG, Dunn’s post hoc test with Bonferroni adjustment was used to compare pairings. Positive mean rank differences indicate that the second group has elevated hormone levels.

## Data Availability

The original contributions presented in this study are included in the article. Further inquiries can be directed to the corresponding authors.
